# The Mitochondrial Chaperone TRAP1 as a Candidate Target of Oncotherapy

**DOI:** 10.3389/fonc.2020.585047

**Published:** 2021-01-26

**Authors:** Shulan Xie, Xuanwei Wang, Shuyuan Gan, Xiaodong Tang, Xianhui Kang, Shengmei Zhu

**Affiliations:** ^1^Department of Anesthesiology, The First Affiliated Hospital, Zhejiang University School of Medicine, Hangzhou, China; ^2^Department of Orthopedics, The First Affiliated Hospital, Zhejiang University School of Medicine, Hangzhou, China

**Keywords:** tumor necrosis factor receptor-associated protein 1, tumorigenesis, metabolic reprogramming, apoptosis resistance, inhibitors, cancer therapy

## Abstract

Tumor necrosis factor receptor-associated protein 1 (TRAP1), a member of the heat shock protein 90 (Hsp90) chaperone family, protects cells against oxidative stress and maintains mitochondrial integrity. To date, numerous studies have focused on understanding the relationship between aberrant TRAP1 expression and tumorigenesis. Mitochondrial TRAP1 is a key regulatory factor involved in metabolic reprogramming in tumor cells that favors the metabolic switch of tumor cells toward the Warburg phenotype. In addition, TRAP1 is involved in dual regulation of the mitochondrial apoptotic pathway and exerts an antiapoptotic effect on tumor cells. Furthermore, TRAP1 is involved in many cellular pathways by disrupting the cell cycle, increasing cell motility, and promoting tumor cell invasion and metastasis. Thus, TRAP1 is a very important therapeutic target, and treatment with TRAP1 inhibitors combined with chemotherapeutic agents may become a new therapeutic strategy for cancer. This review discusses the molecular mechanisms by which TRAP1 regulates tumor progression, considers its role in apoptosis, and summarizes recent advances in the development of selective, targeted TRAP1 and Hsp90 inhibitors.

## Introduction

Molecular chaperones, including heat shock proteins (Hsps), are a class of ubiquitous intracellular proteins. These proteins mediate the correct assembly of other proteins but are not components of the final functional structure. Hsps perform important functions in maintaining protein homeostasis, such as mediating protein folding, assembly, and intracellular localization and regulating apoptosis. Failure of these processes may lead to disease (1).

Tumor necrosis factor receptor-associated protein 1 (TRAP1), a member of the Hsp90 chaperone family, is primarily a mitochondrial matrix protein (2). TRAP1 was initially identified in a yeast two-hybrid screen as a novel protein that binds to the intracellular domain of the type I tumor necrosis factor receptor (3). The mitochondrial chaperone TRAP1 plays an important role in maintaining mitochondrial integrity and intracellular homeostasis and is closely related to apoptosis. To date, the main identified functions of TRAP1 include (i) antagonizing the proapoptotic activity of cyclophilin D (CypD) to subsequently inhibit mitochondrial permeability transition pore (mPTP) opening (4, 5), (ii) reducing reactive oxygen species (ROS) generation to protect cells against oxidative stress (6), (iii) regulating endoplasmic reticulum (ER) stress (7), and (iv) inhibiting the activity of succinate dehydrogenase (SDH), thus regulating mitochondrial bioenergetics (8).

Recently, an increasing number of studies have shown that the mitochondrial chaperone TRAP1 functions as either an oncogene or a tumor suppressor in human malignant tumors and regulates tumor development (9). Cancer is a group of complex diseases with several hallmarks, which are considered phenotypic adaptations to overcome all obstacles during disease progression (10). Among these hallmarks, metabolic reprogramming is required for tumor cell survival (10). TRAP1 is involved in metabolic reprogramming and affects the switch between oxidative phosphorylation (OXPHOS) and aerobic glycolysis (11). In addition, the TRAP1 protein is involved in many other cellular processes. TRAP1 regulates the cell cycle to modulate cell proliferation (12) and promotes tumor metastasis by inducing mitochondrial fission (13). Furthermore, interference with the function of TRAP1 may result in the death of tumor cells but does not affect normal cells (13). Thus, an approach that selectively targets TRAP1 might be a promising strategy for the development of novel antitumor drugs. This review explores the relationship between aberrant expression of TRAP1 and tumorigenesis, including the molecular mechanisms by which TRAP1 regulates tumor progression; considers the role of TRAP1 in apoptosis; and evaluates the potential therapeutic value of TRAP1 in cancers.

## Aberrant Expression of TRAP1 in Tumors

For decades, the expression of TRAP1 has been reported to be closely associated with the occurrence and development of tumors (**Table 1**). TRAP1 expression is upregulated in various human malignancies, including nasopharyngeal (23), breast (14), and prostate (21) cancers, as well as non-small cell lung cancer (16). For example, the first large-scale study of human colorectal adenocarcinoma tissues revealed a statistically significant positive correlation between pathological T stage and TRAP1 expression. This study also proposed that TRAP1 causes tumor cells to invade stromal tissue by inducing epithelial-mesenchymal transition (EMT) (20), which is implicated in the metastasis of primary tumors (15). As shown in a study by Tian et al., TRAP1 is expressed at significantly higher levels in esophageal squamous cell carcinoma (ESCC) tissues than in adjacent normal tissues, and TRAP1 expression is inversely proportional to the degree of ESCC differentiation. TRAP1 knockdown in ESCC cells results in cell cycle dysregulation by inducing G2/M arrest and causes a marked increase in the proportion of apoptotic cells (24). Si et al. observed a substantial increase in TRAP1 expression in kidney cancer tissues compared with normal kidney tissues that was closely related to lymph node metastasis, clinical stage, and patient prognosis. Patients with high TRAP1 expression had a poor prognosis (17). In addition, Wu et al. showed that TRAP1 knockout substantially decreased the proliferation and migration of glioblastoma multiforme cells, induced apoptosis and G2/M arrest, and inhibited neurosphere recovery and secondary neurosphere formation through its regulatory effects on metabolic reprogramming (18). These data indicate that the TRAP1 protein may exert oncogenic effects, promoting the proliferation, invasion, and apoptosis resistance of human malignant tumor cells and affecting the prognosis of patients with cancer.

**Table 1:** Aberrant TRAP1 expression in different tumors.

Lineage	Cancer subtype	No. of samples	TRAP1 expression characteristics	Ref.
Nasopharynx	Nasopharyngeal carcinoma (NPC)	56	Higher expression in NPC tissues than in normal noncancerous nasopharyngeal tissues	(14)
Colorectum	Colorectal adenocarcinoma	714	Positive correlation with the pathological T stage	(15)
Prostate	Prostate cancer	61	High expression in localized and metastatic prostate cancer	(16)
Esophagus	Esophageal squamous cell carcinoma (ESCC)	138	Higher expression in ESCC tissues than in adjacent normal tissues	(17)
Kidney	Kidney cancer	110	Higher expression in kidney cancer than in normal kidney tissues; significantly correlated with lymph node metastasis, the clinical stage, and patient prognosis	(18)
Brain	Glioblastoma multiforme (GBM)	22	Higher expression in GBM specimens than in adjacent noncancer tissues; positive correlation with GBM cell proliferation and migration	(19)
Lung	Non-small cell lung cancer (NSCLC)	71	Inverse correlation with the prognosis in patients with NSCLC	(20)
Breast	Breast cancer	42	Inverse correlation with the metastatic capacity	(21)
Ovary	Epithelial ovarian carcinoma (EOC)	208	Positive correlation with the chemotherapeutic response and overall survival of patients with estrogen receptor α (ERα)-positive tumors	(22)

However, TRAP1 may also function as a tumor suppressor in certain tumor cells. Yoshida et al. reported a significant inverse correlation between TRAP1 expression and tumor stage in patients with cervical cancer, bladder cancer, and clear cell renal cell carcinoma (11). In addition, TRAP1 knockdown was found to increase ROS levels, promote mitochondrial c-Src activation, and substantially increase cell motility and invasion (11), suggesting that cancers with low expression of TRAP1 may be more likely to spread and disseminate from the primary site (19). Furthermore, Aust et al. studied estrogen receptor α-positive ovarian cancer cells and observed significant positive correlations between high TRAP1 expression and estrogen receptor α positivity with the chemotherapeutic response and overall survival (25). In addition, Amoroso et al. documented a high rate of TRAP1 gene deletion in high-grade serous ovarian cancer, and the TRAP1 expression level was associated with the gene copy number, which might partially explain the low TRAP1 expression observed in patients with ovarian cancer (19). In summary, the role of TRAP1 in different types of cancers is related to the disease state and prognosis of patients, suggesting that TRAP1 constitutes a target for tumor treatment.

## Roles of TRAP1 in Cancer Metabolism

Most normal cells mainly generate a large amount of ATP through the OXPHOS under aerobic conditions but obtain energy by glycolysis under hypoxic or anoxic conditions. However, even under conditions with a sufficient amount of oxygen, glycolysis is also active in most malignant tumor cells, which are characterized by a high rate of glucose uptake and high lactic acid content among metabolites. The metabolic characteristics of this shift toward aerobic glycolysis are called the Warburg effect (22), and this reprogramming of energy metabolism is essential for tumor growth, proliferation, and metastasis (10). Several possible mechanisms contribute to the Warburg effect, including the following: (i) Bioenergetics: ATP production through glycolysis (two ATP molecules per glucose molecule) is much less efficient than ATP production through OXPHOS (36 ATP molecules per glucose molecule); thus, cancer cells maintain energy homeostasis by substantially increasing their glycolytic activity (26, 27), which is beneficial to the rapid growth of cancer cells. (ii) Biosynthesis: The growth of tumors is faster than normal tissue; thus it requires not only energy but also biomacromolecules for growth. Glycolysis provides metabolic intermediates and precursors for macromolecular biosynthesis and then promotes the production of NADPH, ribose 5-phosphate, and nonessential amino acids, thus contributing to the biosynthesis of nucleic acids, lipids, and proteins (28). (iii) Hypoxia: The unlimited proliferation of tumor cells results in the disorder of tumor vascular architecture, the defect of self-regulation ability, and the change of hemorheology thus leads to the hypoxia of local tumor tissue. Thus, the glycolysis may be increased in cancer cells for adaptation to the hypoxic environment (26). Hypoxia-inducible factor-1 (HIF-1) is a key regulator of hypoxia adaptation in tumor cells, which increases tumor glycolytic activity and angiogenesis (29). (iv) Microenvironment acidification: Lactic acid accumulation caused by a high glycolytic rate results in acidification of the tumor microenvironment, which facilitates invasion *via* extracellular matrix breakdown and inhibits the immune response to tumor antigens (30). In summary, to adapt to the surrounding microenvironment and compete with surrounding normal cells for limited resources such as glucose, cancer cells shift their metabolic characteristics toward the Warburg phenotype to preserve their growth. Cancer cells acquire and metabolize nutrients in a way that facilitates proliferation rather than efficient production of ATP.

As a key regulatory factor in tumor metabolic reprogramming, TRAP1 is involved in a variety of mechanisms. TRAP1, as a Myc target gene, maintains the folding and stability of mitochondrial OXPHOS complexes II (SDHB) and IV (cytochrome c oxidase II), promoting the growth of tumors (31). Under metabolic stress conditions, particularly nutrient-poor and hypoxic conditions, SDHB regulation with Hsp90/TRAP1-directed protein folding produces sufficient energy to meet the energy metabolism requirements of tumor cells (32). The mitochondrial chaperone TRAP1 competitively binds to SDHA and then decreases SDH activity, resulting in suppression of mitochondrial respiration and an increase in the intracellular succinate concentration. Even under normoxic conditions, succinate accumulation stabilizes HIF-1α and then induces pseudohypoxia (8, 33). HIF-1 not only contributes to the switch of tumor cell metabolism to the Warburg phenotype but also regulates EMT, angiogenesis and other processes to promote the occurrence and development of tumors (34). Thus, TRAP1 results in accumulation of HIF-1α, inhibits mitochondrial respiration and promotes the switch of energy metabolism from OXPHOS to aerobic glycolysis through its interaction with SDH. Consistent with the data described above, Yoshida demonstrated that TRAP1 deficiency increased mitochondrial respiration and the levels of tricarboxylic acid cycle intermediates. The authors identified TRAP1 as a crucial regulator of the metabolic switch of tumor cells between OXPHOS and aerobic glycolysis, which correlated with the interaction of TRAP1 and mitochondrial c-Src (11). Mitochondrial c-Src modulates mitochondrial respiration and ROS generation by phosphorylating respiratory chain components (35).

However, a study reevaluated the flux control and distribution of OXPHOS and glycolysis in tumor cells and proposed that not all types of tumor cells use aerobic glycolysis as their primary metabolic pathway (36). As shown in a study by Pastò et al., epithelial ovarian cancer cells depend mainly on OXPHOS rather than glycolysis to maintain tumor growth, indicating that epithelial ovarian cancer cells escape the Warburg effect (37). As ovarian cancer progresses, the expression of TRAP1 decreases, the cellular metabolic characteristics shift to OXPHOS, and both the invasion and cisplatin resistance of the cancer cells increase (38). Consistent with the data described above, a recent study indicated a dual function of TRAP1 as both an oncogene and a tumor suppressor in human tumors; these functions are driven by different histotype-specific energy metabolism characteristics (9). Thus, aberrant expression of TRAP1 in human malignant tumors is closely correlated with its role in metabolic reprogramming. The identification of TRAP1 as a critical factor regulating the metabolic switch of tumors might provide new insights into cancer therapy.

## Roles of TRAP1 in Apoptosis Resistance

**Figure 1:**
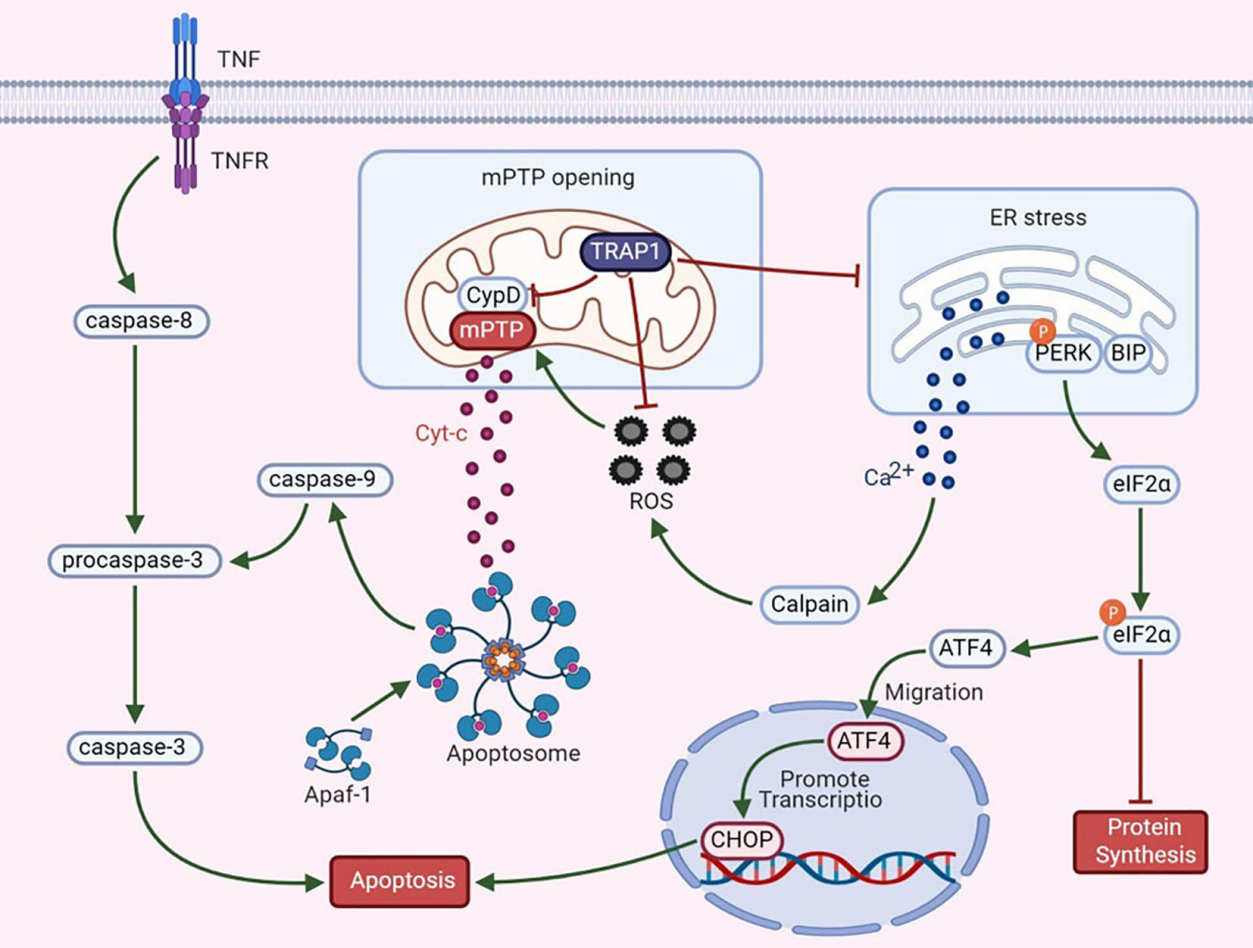
The dual regulatory effects of TRAP1 on the mitochondrial apoptotic pathway.

Apoptosis is regulated by proteins expressed by certain evolutionarily conserved genes. Hsp90 is highly conserved from bacteria to mammals and is closely associated with apoptosis. During apoptosis, an interaction between mitochondria and the ER involves the fusion of these two organelles, and the resulting regions are called mitochondria-associated ER membranes (MAMs). MAMs participate in the coordination of Ca^2+^ signal transduction, triggering apoptosis (39, 40). TRAP1 exerts dual regulatory effects on the mitochondrial apoptotic pathway (**Figure 1**).

Mitochondrial permeability transition, which is regulated mainly by the mPTP, is a key mechanism in apoptosis. An increase in mPTP opening causes the release of cytochrome c (Cyt-c), widespread swelling of mitochondria and loss of the mitochondrial membrane potential (41). The release of Cyt-c is an important signal of mitochondrial apoptosis and activates apoptotic protease-activating factor 1 and procaspase-3. Cyt-c and deoxyadenosine triphosphate (dATP) interact to form the apoptosome. Activation of caspase-3 *via* its cleavage further inhibits the expression of poly (adenosine diphosphate-ribose) polymerase, thus triggering the apoptotic cascade (42, 43). Furthermore, CypD, a cyclosporin A-binding protein located in the mitochondrial matrix, is a component and a key regulator of the mPTP to maintain mitochondrial integrity. CypD and the mPTP play essential roles in the mechanism regulating mitochondrial Ca^2+^ homeostasis. Excess mitochondrial Ca^2+^ binds to the F1 domain of F1F0 ATP synthase, alters the synthesis of ATP, increases the release of cytotoxic free radicals, and causes CypD-mediated opening of the mPTP, thus triggering cell death (44, 45). Overexpression of TRAP1 blocks the mitochondria-mediated apoptotic cascade to prevent apoptosis, as manifested by the inhibition of CypD-dependent mPTP opening, a reduction in mitochondrial Cyt-c release, and a decrease in caspase-3 activity (4, 5). According to Xiang et al., under hypoxic conditions, TRAP1 overexpression in cardiomyocytes exerts a protective effect by regulating the opening of the mPTP (46). In a study by Liu et al., TRAP1 was found to decrease high glucose-induced apoptosis, increase cell viability, ameliorate mitochondrial damage, and improve renal function in diabetic rats by blocking mPTP opening (47). Based on these findings, TRAP1 antagonizes the proapoptotic effect of CypD to regulate mPTP opening, thus playing an antiapoptotic role.

Mitochondria are the main source of ROS *in vivo*. In the presence of oxidative stress, intracellular redox homeostasis is disrupted, leading to the accumulation of ROS. ROS, effective inducers of oxidative damage, cause general cellular damage by inducing the oxidation of proteins, lipids, polysaccharides, and DNA (48). TRAP1 reduces the generation of ROS and superoxide anions by inhibiting SDH and alters redox equilibrium to protect malignant cells against oxidative stress (8). TRAP1 interference induces ROS accumulation, whereas TRAP1 overexpression antagonizes ROS production (6). Overproduction of ROS is related to mitochondrial Ca^2+^ overload and Cyt-c release, subsequently triggering mPTP opening and cell death (49). As shown in a study by Zhang et al., during simulated myocardial ischemia/reperfusion, overexpression of TRAP1 prevents apoptosis by decreasing ROS generation and delaying mPTP opening (50). These data indicate that TRAP1 protects cells against oxidative damage and apoptosis by antagonizing ROS generation. However, nuclear ROS induce DNA damage and mutation, leading to genomic instability and promoting tumor progression (51). This observation suggests that TRAP1 may inhibit the development of certain tumors.

Based on accumulating evidence, the ER also functions as a critical apoptosis control point (52). The ER is the main site of protein synthesis, folding and transport. Many stress conditions, including the destruction of intracellular homeostasis and changes in the oxidative environment in the ER, induce ER stress. The UPR is a signaling pathway that restores ER function after ER stress activation; in addition, it interacts and interconnects with various signaling pathways. However, under conditions of excessive or irreversible ER stress, the UPR triggers apoptosis (53). The antiapoptotic activity of TRAP1 also relies on its functions in preventing ER stress and in the quality control of specific mitochondrial client proteins, which is relevant to its regulation of the mitochondrial apoptotic pathway (7). For example, in the ER, TRAP1 interacts with the regulatory proteasomal protein TBP7, which is involved in the quality control of mistargeted/misfolded mitochondria-destined proteins (54). Moreover, the Ca^2+^-binding protein Sorcin exerts a specific effect on mitochondria that is closely related to the antiapoptotic activity of ER-associated TRAP1 and maintains the stability of TRAP1 (55). Takemoto et al. proposed that downregulation of mitochondrial TRAP1 expression increases the expression of ER-resident caspase-4, which is activated by ER stress, to regulate the UPR pathway in the ER (56). Moreover, TRAP1 knockout increases the expression of glucose-regulated protein 78 (GRP78) mRNA and decreases the expression of C/EBPb and C/EBP homologous protein (CHOP) in a time-dependent manner (56). Matassa et al. examined TRAP1 knockdown cells and observed decreased activation of protein kinase RNA-like ER kinase (PERK), an ER membrane-localized sensor kinase, and a transient decrease in the level of phosphorylated eIF2α (57). GRP78 is an ER chaperone and a central regulator of the UPR that protects cells against ER stress-induced apoptosis (58). Phosphorylation of eIF2α is the initial response to ER stress; this phosphorylation step prevents eIF2α from initiating protein translation and reduces global protein synthesis in cells (57). Continuous blockade of protein translation induces cell death. Thus, inhibition of TRAP1 enhances ER stress. Taken together, these observations indicate that TRAP1 may modulate the UPR in the ER. Its role in mitochondria suggests that TRAP1 is involved in tumor cell apoptosis to promote tumor progression.

## The AKT/mTOR/p70S6K Signaling Pathway Participates in TRAP1-Mediated Regulation of Tumor Invasion and Metastasis

**Figure 2:**
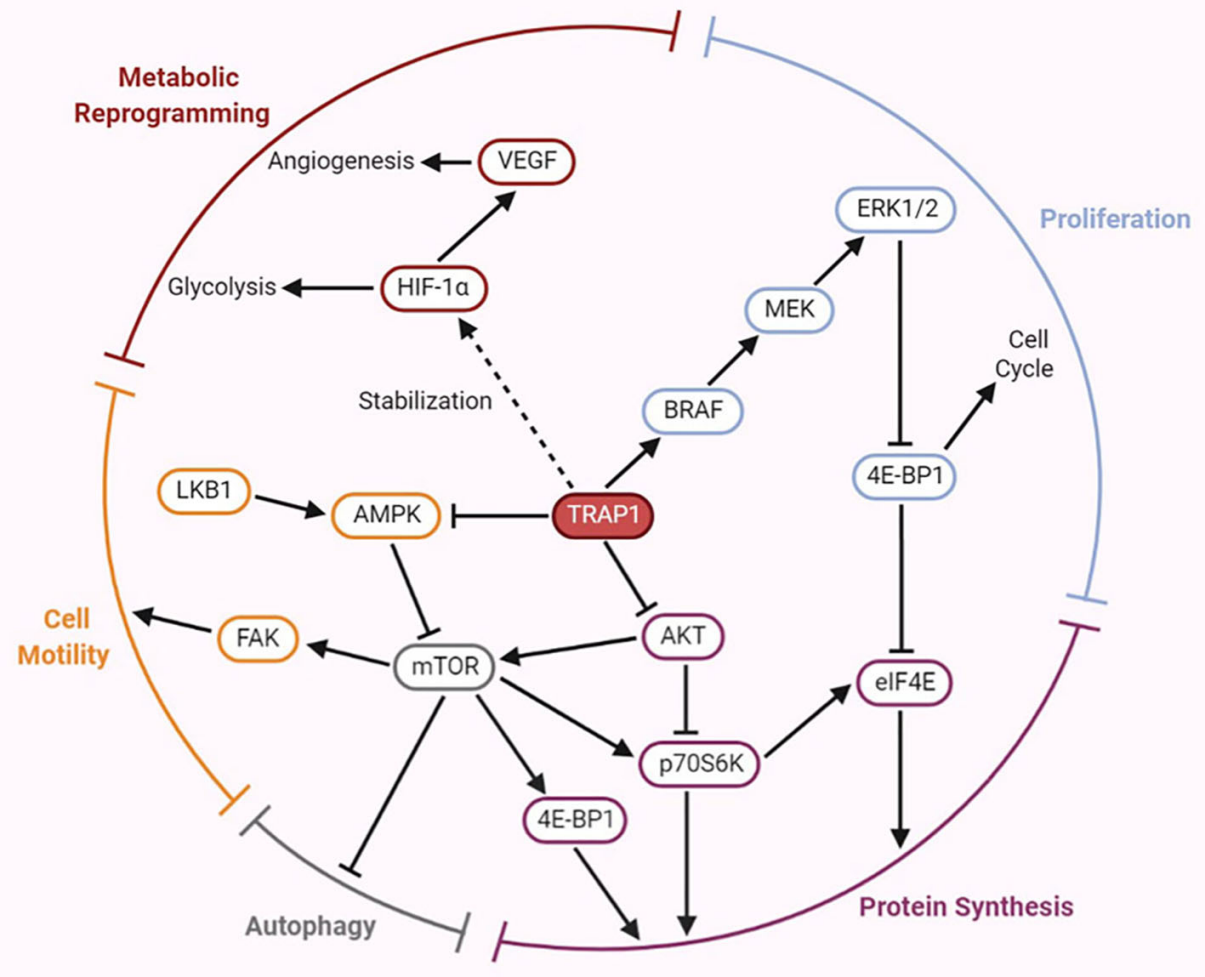
Emerging roles of TRAP1 in tumor development and progression mediated by the regulation of several crucial factors involved in the signaling pathways.

AKT, also called protein kinase B (PKB), directly activates mammalian target of rapamycin (mTOR). Both mTOR and ribosomal protein S6 kinase (p70S6K) are cytoplasmic Ser/Thr protein kinases, and the former activates the latter. The AKT/p70S6K pathway is involved in regulation of the actin cytoskeleton, which is important for cell migration (59). In both normoxia and hypoxia, lack of TRAP1 expression is associated with expression of genes involved in metastasis (12). Similarly, the involvement of TRAP1 in the attenuation of protein synthesis results in an inverse correlation between TRAP1 expression and p70S6K expression (60). Low TRAP1 expression and high p70S6K expression increase cell motility and promote tumor cell migration (19). However, Agliarulo et al. indicated that the effects of TRAP1 on cell motility are partially but not completely related to the AKT/p70S6K pathway, which does not directly affect the actin cytoskeleton or cell-matrix adhesion. p70S6K and glutamine endow TRAP1 knockdown cells with increased migratory potential, while TRAP1 enables cancer cells to migrate even under conditions of nutrient deprivation (61). In addition, previous investigations have shown that the expression of matrix metalloproteinases (MMPs), a family of zinc-dependent endopeptidases—particularly that of MMP2 and MMP9—is induced by the PI3K/AKT signaling pathway (62). The major function of MMPs is to degrade various protein components of the extracellular matrix; thus, they disrupt the histological barrier to tumor cell invasion and play crucial roles in tumor invasion and metastasis (63). Low expression of TRAP1 was found to induce upregulation of MMP2 and MMP9 mRNAs expression in ovarian cancer cells, thus triggering EMT. However, in ovarian cancer, the mechanisms by which TRAP1 induces EMT to regulate cell migration are at least partially independent of the p70S6K pathway (19), consistent with the results of the above mentioned study by Agliarulo et al. Furthermore, activation of the PI3K/AKT/mTOR signaling pathway upregulates the expression of HIF-1α (64), thus triggering the transcription of vascular endothelial growth factor (VEGF) mRNA and increasing VEGF expression (65), which in turn enables endothelial cells to migrate to form new blood vessels and increase the blood supply to tumor cells. These results indicate that TRAP1 signaling partially interacts with the AKT/mTOR/p70S6K signaling pathway to regulate the invasion and metastasis of cancer cells; however, the specific mechanism linking these pathways remains to be studied (**Figure 2**).

## The BRAF/ERK Signaling Pathway Participates in TRAP1-Mediated Regulation of Tumorigenesis

The B-type RAF proto-oncogene (BRAF) is located on human chromosome 7 and encodes a member of the RAF family of Ser/Thr protein kinases. The BRAF protein regulates the MAPK/extracellular signal-regulated kinase (ERK) signaling pathway, affecting cell proliferation, differentiation, and secretion (66). BRAF is one of the 12 most frequently mutated genes and exhibits cancer subtype-specific mutation patterns (67). A recent study proposed an interaction between TRAP1 and BRAF, and TRAP1 was identified as a downstream effector of the cytoprotective BRAF pathway in mitochondria. Activation of the BRAF pathway increases Ser phosphorylation of TRAP1, which may contribute to its antiapoptotic activity. TRAP1-dependent BRAF activation antagonizes apoptosis by inhibiting mPTP opening (68). Liang et al. suggested that both the rate of TRAP1 positivity and the rate of BRAF^V600E^ mutation were increased in papillary thyroid cancer (PTC) and that high TRAP1 expression was closely correlated with the BRAF^V600E^ mutation, both of which may mediate the development of PTC (69). Moreover, results of *in vitro* and *in vivo* studies of TRAP1 in human breast and colorectal carcinoma indicated that TRAP1 is involved in the mechanism regulating the synthesis/ubiquitination of BRAF but does not influence its stability (70). The BRAF level is decreased upon TRAP1 interference, which reduces ERK phosphorylation, inhibits cell cycle progression, and results in accumulation of cells at the G0-G1 and G2-M checkpoints (70). In addition, TRAP1 maintains ERK1/2 activity as a mitochondrial chaperone; in turn, ERK-dependent phosphorylation of TRAP1 inhibits SDH, which is conducive to shifting the metabolic characteristics of tumor cells toward the Warburg phenotype (71). Thus, the BRAF/ERK signaling pathway participates in TRAP1-mediated regulation of cell proliferation and metabolism (**Figure 2**).

## The AMPK/ULK1 Signaling Pathway Participates in TRAP1-Mediated Regulation of Metastasis

AMP-activated protein kinase (AMPK) is a critical molecule regulating bioenergetic metabolism that is necessary for internal glucose homeostasis. Laker et al. were the first to discover that acute exercise induces mitochondrial autophagy through AMPK-dependent activation of UNC-51-like kinase (ULK1) in skeletal muscle to resist mitochondrial oxidative damage induced by high levels of acute exercise (72). Therefore, the AMPK/ULK1 signaling pathway is involved in mediating mitochondrial autophagy, thereby affecting mitochondrial homeostasis. AMPK phosphorylation is inversely correlated with the motility of both tumor and normal cells. TRAP1 inhibits the activation of AMPK and its substrate ULK1, maintains cytoskeletal dynamics, releases the cell motility effector FAK by limiting autophagy during bioenergetic stress, further overcomes metabolic stress and promotes tumor cell metastasis (73). The results described above indicate that TRAP1 is involved in the AMPK-related energy sensing pathway to facilitate tumor cell metastasis (**Figure 2**).

## Development of TRAP1 Inhibitors

To date, TRAP1 inhibitors, including gamitrinibs, shepherdin, DN401, and honokiol bis-dichloroacetate (HDCA), have been developed (**Table 2**). However, no TRAP1-targeted inhibitor has entered the market as an antitumor drug.

**Table 2:** The inhibitors partially or completely acting on TRAP1.

Drug	Structure	Properties	Types of cancer	Refs.
Gamitrinib-G4	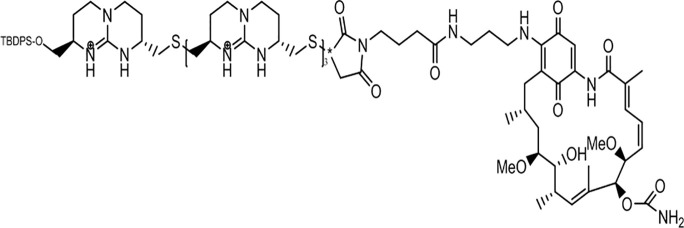	Hsp90 inhibitor and TRAP1 inhibitor	Prostate cancer	(74)
Shepherdin	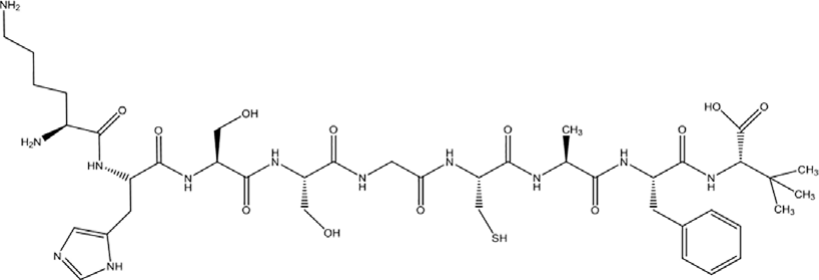	Hsp90 inhibitor and TRAP1 inhibitor	Glioblastoma, retinoblastoma	(75, 76)
DN401	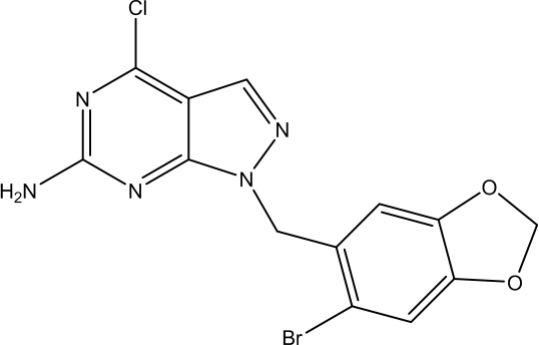	Strong TRAP1 inhibitor but weak Hsp90 inhibitor	Cervical cancer, liver cancer, brain cancer, kidney cancer, lung cancer, prostate cancer	(77, 78)
Honokiol bis-dichloroacetate (HDCA)	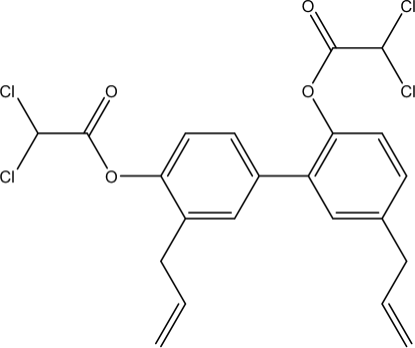	TRAP1-specific inhibitor		(79)

### Inhibitors Simultaneously Acting on Both Hsp90 and TRAP1

Due to the lack of chemical tools for targeted selection of Hsp90 and TRAP1, the existing inhibitors simultaneously act on Hsp90 and TRAP1 and most of them have lower TRAP1 selectivity. Gamitrinibs (geldanamycin mitochondrial matrix inhibitors) and shepherdin are inhibitors of mitochondrial Hsp90 and TRAP1 that do not upregulate Hsp70 expression; these agents mediate cell death *via* CyD-dependent mPTP opening (80). Gamitrinibs contain two main regions: a 17-AAG region and a mitochondrial targeting sequence of either one to four tandem repeats of cyclic guanidinium (gamitrinib-(G1-G4)) or a triphenylphosphonium (gamitrinib-TPP) (81). Gamitrinib-G4 limits the formation of localized and metastatic prostate cancer and showed favorable tolerability in a mouse model of transgenic adenocarcinoma of the prostate (TRAMP). It was a feasible long-term systemic treatment for TRAMP mice, with no evidence of weight loss or organ toxicity in the mice and no effect on prostatic intraepithelial neoplasia or prostatic inflammation (82). In glioblastoma cells, treatment with shepherdin, a peptidomimetic that inhibits the Hsp90-Survivin interaction, causes irreversible mitochondrial cleavage, degradation of Hsp90 client proteins in the cytoplasm, and tumor cell death through apoptosis and autophagy (83). Similarly, shepherdin treatment of retinoblastoma cells decreases the stability of Survivin, decreases the activity of MMP-2, and increases the expression of the proapoptotic proteins Bax, Bim, and caspase-9, which induces caspase-dependent apoptosis and autophagy (84). Besides, DN401 is a derivative of the purine scaffold of the Hsp90 inhibitor BIIB-02112 with strong TRAP1 binding affinity but weak Hsp90 binding affinity (85). DN401 degrades client proteins of Hsp90 and TRAP1 without inducing Hsp70 in different cancer cells; this mechanism increases mitochondrial fragmentation and cell apoptosis, thus enhancing the anticancer activity of the drug (86).

### TRAP1-Specific Inhibitors

At present, many studies have been devoted to the specific inhibitors which act on TRAP1 but not Hsp90. Inhibitors that selectively inhibit the function of TRAP1 but not other Hsp90 proteins could positively affect the treatment of TRAP1-dependent diseases and provide new insight into antitumor strategies. HDCA is a novel TRAP1-specific inhibitor with antitumor activity. By binding an allosteric site in TRAP1, HDCA inhibits TRAP1 but not Hsp90 ATPase activity in a concentration-dependent manner to enhance SDH activity, thus decreasing tumor cell proliferation and tumorigenic growth (87). Rondanin et al. envisaged a potential strategy for inducing apoptosis by targeting the TRAP1 ATPase domain, with cationic appendages selected as carriers for drug delivery to the mitochondria. This group confirmed that the accumulation of guanidine-based compounds in mitochondria inhibited the expression of recombinant TRAP1 ATPase, thus limiting the proliferation and inducing the apoptosis of colon carcinoma cells (88). Sanchez-Martin et al. reported that structure- and dynamics-based allosteric ligands for selective targeting of TRAP1 could be considered a novel therapeutic strategy for cancer. This recent study provides additional prospects for the development of TRAP1-specific inhibitors (89). How to design a novel tool for efficient selection of TRAP1 site is a problem that needs to be solved.

## Anticancer Potential of TRAP1 Inhibitors Combined With Chemotherapeutics

TRAP1 upregulation protects against oxidative stress-/cisplatin-induced DNA damage and apoptosis (90). TRAP1 overexpression prevents HT-29 colorectal cancer cells from undergoing apoptosis induced by 5-fluorouracil, oxaliplatin, and irinotecan, while shepherdin overcomes the resistance to these chemotherapeutic drugs by inhibiting the TRAP1 ATPase (91). As shown in a study by Kuchitsu et al., TRAP1 interference reduces the proliferation of lung adenocarcinoma cells and increases their sensitivity to cisplatin, indicating that TRAP1 expression may affect recurrence and chemotherapeutic resistance in patients with lung adenocarcinoma (74). These results indicate that TRAP1 increases tumor resistance to chemotherapeutic drugs. Based on the role of TRAP1 in promoting a multidrug-resistant phenotype, combined targeting of TRAP1 and treatment with chemotherapeutic drugs may exert synergistic anticancer activity toward a broad range of human malignant tumors. The purpose of combination therapy is to improve the therapeutic effect and avoid unwanted side effects (75). Taken together, these findings indicate that TRAP1 inhibitors treatment combined with chemotherapy may become a new therapeutic strategy for cancer. At present, most of the studies have focused on the combination of Hsp90 inhibitors and chemotherapy drugs (76–79), so further studies are required to the synergistic effect of TRAP1-specific inhibitors and chemotherapy.

## Concluding Remarks and Future Perspectives

TRAP1, a molecular chaperone that is ubiquitous and abnormally expressed in tumors, has recently become the focus of numerous studies as an oncotherapeutic target. A close association between TRAP1-dependent regulation of metabolic characteristics and the role of TRAP1 in cancer progression has been identified. Thus, the functions of mitochondrial TRAP1 in both normal and tumor cells are likely more complex than previously recognized. In addition, TRAP1 can promote the proliferation, increase the motility, and facilitate the invasion and metastasis of tumor cells by regulating several signaling pathways. Selective TRAP1-targeted inhibitors have facilitated the development of antineoplastic drugs, and several drugs with targeted selectivity for Hsp90 and TRAP1 have been developed. These drugs have shown good tolerability, strong cytotoxic activity, and no organ toxicity in preclinical studies. The efficiency of TRAP1 inhibitors combined with chemotherapeutic agents for cancer treatment is being investigated in clinical trials. Due to the lack of chemical tools for targeted selection of Hsp90 and TRAP1, additional studies are needed to improve TRAP1 selectivity and achieve improved therapeutic effects. However, researchers have not yet determined whether prolonged inhibition of Hsp90 induces drug resistance (92). Suitable *in vitro* and *in vivo* models must be established to elucidate the mechanisms of TRAP1 and to develop specific inhibitors.

## Author Contributions

XK and SZ contributed design of the study and manuscript editing. SX wrote the first draft of the manuscript. XW, SG, and XT helped prepare the manuscript and collect the data. All authors contributed to the article and approved the submitted version.

## Funding

This research was supported by the Natural Science Foundation of China under Grant No.81971008 and Zhejiang Provincial Natural Science Foundation of China under Grant No.LY19H310009.

## Conflict of Interest

The authors declare that the research was conducted in the absence of any commercial or financial relationships that could be construed as a potential conflict of interest.
